# Swelling Mechanisms of UO_2_ Lattices with Defect Ingrowths

**DOI:** 10.1371/journal.pone.0134500

**Published:** 2015-08-05

**Authors:** Seçkin D. Günay

**Affiliations:** Yıldız Technical University, Department of Physics, Faculty of Science, Esenler, Istanbul, Turkey; University of Akron, UNITED STATES

## Abstract

The swelling that occurs in uranium dioxide as a result of radiation-induced defect ingrowth is not fully understood. Experimental and theoretical groups have attempted to explain this phenomenon with various complex theories. In this study, experimental lattice expansion and lattice super saturation were accurately reproduced using a molecular dynamics simulation method. Based on their resemblance to experimental data, the simulation results presented here show that fission induces only oxygen Frenkel pairs while alpha particle irradiation results in both oxygen and uranium Frenkel pair defects. Moreover, in this work, defects are divided into two sub-groups, obstruction type defects and distortion type defects. It is shown that obstruction type Frenkel pairs are responsible for both fission- and alpha-particle-induced lattice swelling. Relative lattice expansion was found to vary linearly with the number of obstruction type uranium Frenkel defects. Additionally, at high concentrations, some of the obstruction type uranium Frenkel pairs formed diatomic and triatomic structures with oxygen ions in their octahedral cages, increasing the slope of the linear dependence.

## Introduction

Uranium dioxide (UO_2_) is primarily used as a nuclear fuel, usually under extreme conditions. For instance, it is exposed to corrosive and radioactive environments at operating temperatures of up to 2000°C. UO_2_ has been widely studied in order to understand its thermo physical, transport, and defect properties [1–14]. All of these properties have been comprehensively summarized by Govers et al. 2007 [15,16]. Molecular dynamics simulations of temperature dependent physical properties of UO_2_ have been used to assist ongoing experiments, which cannot be done easily because of the required extreme conditions. Such properties include lattice parameter, volume, density, electrical resistivity, and diffusion and they are sensitive to both temperature and irradiation [17–22]. Radiation damage to UO_2_ crystals greatly influences reactor fuel, which in turn degrades performance.

Heavy ions, fission products, alpha particles, alpha-recoil atoms (alpha particles and recoil nuclei produced by alpha decay), and neutrons cause irradiation damage. Moreover, electrons, X-rays, and gamma rays intensify damage but are generally ignored [17]. When a UO_2_ crystal is exposed to radiation, Frenkel pair (FP) defects are created along the direction of irradiation. A FP results when radiation causes an atom to be displaced from its regular lattice position, producing a lattice vacancy and an interstitial atom. Furthermore, if the radiation dose is sufficiently high, complete amorphization may occur at ambient conditions [17]. Experimentally, Nakae et al. [18–20] and Weber [21,22] have studied fission and α-particle dose effects, as well as the temperature dependences and recovery behaviors of the lattice parameter and lattice strain of irradiated UO_2_.

Molecular dynamics simulation studies on radiation damage have previously investigated defect production and clustering by energetic uranium recoils in UO_2_ [23]. In addition, Aidhy et al. [24] have explored the kinetic evolutions of irradiation-induced point defects in UO_2_ at 1000 K using molecular dynamics simulations. They observed that if such defects are present in only one sublattice, FPs recombine during equilibration, but if defects are present in both sublattices, they form clusters. They concluded that the radiation tolerance of a material is primarily determined by its cation sublattice. However, to the best of our knowledge, to date, the lattice swelling that result from defect ingrowth and the temperature dependence of the lattice recovery of defected UO_2_ have not been simulated.

In the present study, molecular dynamics calculations were carried out on UO_2_ supercells. Two different types of partially ionized rigid ion potentials, taken from the literature [1,5], were used for the interionic interactions. The ingrowth of defects was investigated in relation to lattice swelling and the results are compared with experimental data.

## Molecular Dynamics Simulations

### Procedure

Crystalline uranium dioxide, with four uranium and eight oxygen ions in its unit cell, has a fluoride type structure. Uranium ions reside at the center of the cubic structure and are coordinated to eight oxygen anions. Oxygen ions are surrounded by four uranium ions. The molecular dynamics (MD) cell was constructed of 500 cations and 1000 anions in a 5×5×5 array of supercells in five mutually orthogonal directions. Calculations were carried out using the MD code Moldy [25]. Long-range coulomb interactions were accounted for using Ewald’s summation [26]. The positions and velocities of the ions were calculated by Beeman’s algorithm, which is of the predictor-corrector type, using a time step Δ*t* = 1.0 fs. The system was simulated at constant pressure and temperature (NPT) at 300 K using the Nose-Hoover thermostat and Parinello-Rahman constant stress methods. Equilibrium runs were performed for 10 ps and data were collected for the following 40 ps.

### Sample preparation

**Fig 1:**
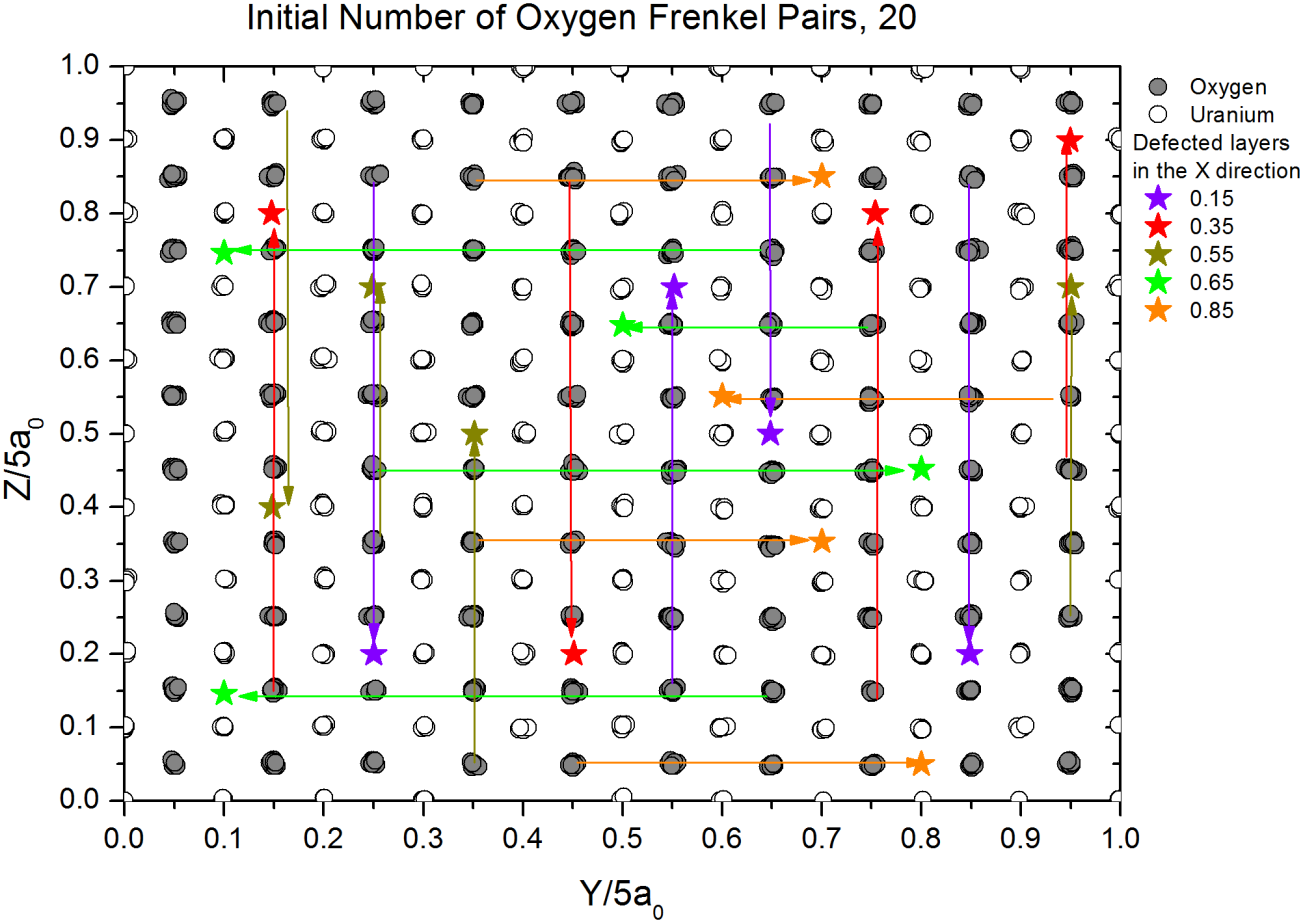
Initial view from the x direction of a MD supercell sample with 20 oxygen IFPs. Arrows indicate the displacements of atoms from their lattice sites to their interstitial positions. Each color represents a different layer along the x direction.

5×5×5 supercells of irradiated samples with different defect concentrations were prepared by randomly moving ions from lattice sites to interstitial positions within the layers. A representative supercell with such defects is shown in Fig 1. Hereafter, these ions will be referred to as initial Frenkel pair (IFP) defects and those present after equilibration will be referred to as Frenkel pair (FP) defects. To minimize annihilation effects, IFP defects were not included in successive layers, so that vacancies and interstitials did not directly terminate one another. Each defect layer has approximately the same number of IFPs. The same simulation procedure was used for both defected and perfect supercell boxes. Uranium dioxide supercells with several different IFP concentrations, based on dose experiments [17], were prepared. In order to correlate the sublattice (oxygen or uranium sublattice) effects with irradiation type, supercells were constructed with either oxygen IFPs or uranium IFPs but not both. Numbers of semi-empirical potentials have been developed in order to model the interactions between the ions of UO_2_ [15]. Most of these potentials parameterized with respect to lattice constant, bulk modulus and/or elastic constants satisfactorily estimate the thermophysical properties of UO_2_ at low temperature but do not able to reproduce the lattice properties at high temperatures at solid phase as well as liquid phase.

There are two potential models of Buckingham-Morse and Vashishta-Rahman types in literature parameterized by Yakub and Günay, respectively, that solid and liquid properties and phase transitions of UO_2_ have been reasonably reproduced. Despite the limitations of any classical potential, namely electronic structure can not be investigated, it can be extremely useful to study atomic structure and defect dynamics of a system containing large number of particles as the quantum mechanical simulation technique is carried for a relatively small number of particles. Studying the swelling mechanism of the lattices with defect ingrowths requires a potential model which reproduces defect properties and large number of particles. Therefore, interionic interactions were modeled using two different types of rigid ion pair potentials, one parameterized by Yakub et al. [5], and the other by Günay et al. [1] where those have been shown to be successful to study defect properties for wide range of temperatures.

Additional calculations were performed for uranium IFPs using an enlarged cell, 8×8×8 unit cells, and with a longer simulation time, 100 ps. Results were compared with those obtained from 5×5×5 supercells. Data were recalculated using a bigger supercell because defects might influence periodic defect images where uranium IFP simulations produce too many defects.

### Calculation of number of defects

**Fig 2:**
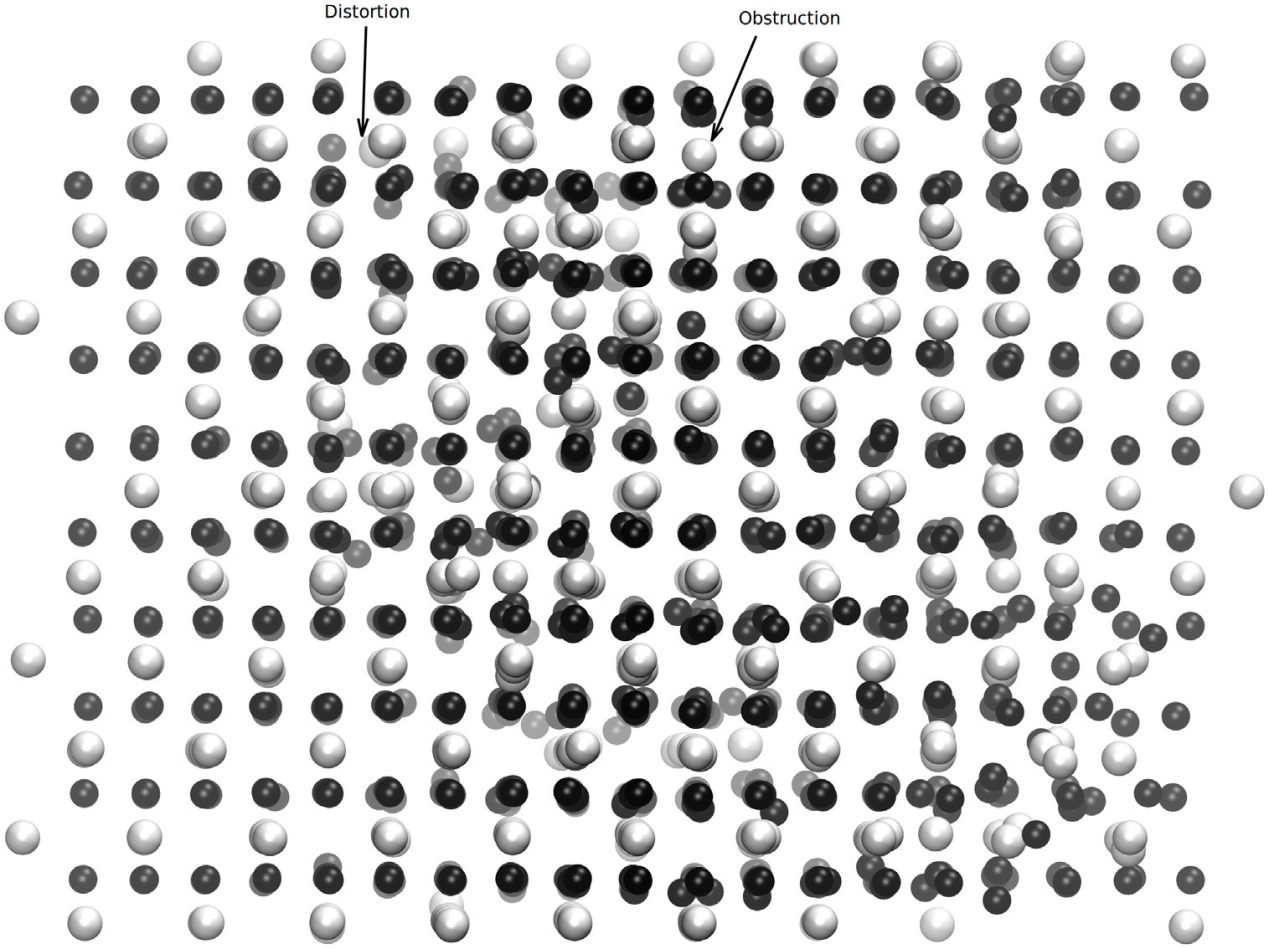
Equilibrium state from the <110> direction of a UO_2_ supercell with 17 uranium IFPs seen. Distortion and obstruction type uranium (gray) defects are indicated with arrows. Distortion type defects are ions that have been slightly displaced into channels. Obstruction type defects are ions that occupy central positions within channels; they occupy special positions, have a constant coordination number, and can be considered to be ions trapped in cages. There is a relationship between the ingrowth of obstruction type defects and radiation-induced lattice expansion.

Here, we describe how the numbers of oxygen and uranium FPs in the prepared samples were calculated. Although it is possible to visually determine numbers of defects using the VMD program [27], as can be seen from Fig 2, we have developed a method for calculating average FP numbers. The results obtained from this method were consistent with visual observations.

**Fig 3:**
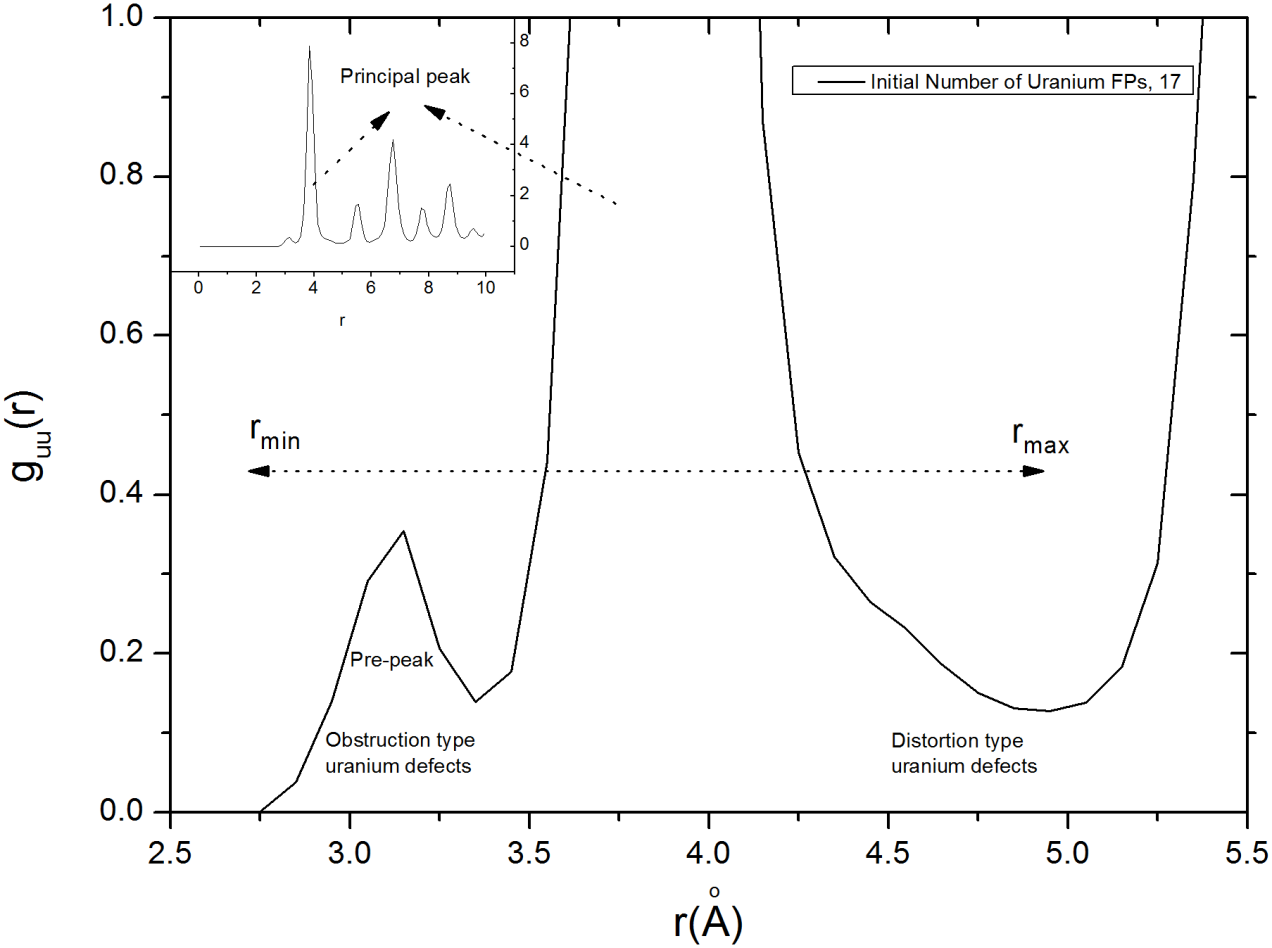
Radial distribution function, *g*_*uu*_(*r*), of a 5×5×5 MD UO_2_ supercell with 17 uranium IFPs. Obstruction and distortion type defects are indicated on the graph. Obstruction type defects have sharper boundaries (pre-peaks) than distortion type defects.

In this method, the number of defects, $\bar{M_{\alpha }}$, is calculated by averaging the defect numbers over the number of time steps, *n*_*t*_, using the following relationship,

$$\bar{M_{\alpha }}=\frac{\sum_{i=1}^{n_{t}}\sum_{j=1}^{N_{\alpha }}K_{ij}}{n_{\alpha \beta }n_{t}}$$ (1)

where *K*_*ij*_ represents the number of *β* ions around the *j* ^*th*^ *α* ion during the *i* ^*th*^ time step within the range Δ*r* = *r*_max_-*r*_min_ and *n*_*αβ*_ is the first coordination number of the perfect crystal *n*_*uu*_ = 12, *n*_*oo*_ = 6, *n*_*uo*_ = 8. The *r*_min_ and *r*_max_ values are determined from the first peak of the radial distribution function, *g*_*αβ*_(*r*), constrained such that $\bar{M_{u}}≅N_{u}=500$ and $\bar{M_{o}}≅N_{o}=1000$. As a representative example, *g*_*uu*_(*r*), used to determine the maximum and minimum values of *r*, is given in Fig 3. Distortion and obstruction type defects (see Fig 2) can, respectively, be considered to be displaced ions in channels and ions that occupy the center positions of channels. An obstruction type ion has a constant number of ions surrounding it which possess a particular position but a distortion type ion is much more mobile which has an indefinite position. Fig 3 shows a distinct pre-peak, just before the principal peak, that reflects the distribution of obstruction type FPs.

Eq (1) gives the average number of obstruction type defects; ${\bar{M_{u}}}^{obs.}≅14$ for the range Δ*r*≅3.4–0.0. To calculate the distortion types of FPs, Δ*r* is determined by assuming the principal *g*_*αβ*_(*r*) peak to be symmetrical. Determinations of distortion type defect Δ*r* intervals are somewhat ambiguous compared to those of obstruction type defects. Here again, a radial distribution function is helpful. ${\bar{M_{u}}}^{dist.}$ has been calculated to be approximately 21 for the range Δ*r*≅4.947–4.5. These calculated defect numbers are consistent with those determined by visual inspection of the VMD snapshot shown in Fig 2.

## Results and Discussions

### Lattice swelling due to defect ingrowth

The lattice expansion that occurs as a result of alpha particle damage is several orders of magnitude greater than that occurs as a result of fission damage [17]. Relative lattice expansions, Δ*a*/*a*_*o*_, were calculated using IFP defect numbers of up to 40, for the temperature of 300 K, and 5×5×5 supercell. Comparing calculated relative lattice expansion results with those obtained from experiment indicated that the calculations carried out on oxygen IFPs corroborated fission experiments and those carried out on uranium IFPs corroborated alpha particle experiments. Therefore, the oxygen and uranium results will be presented in two separate sections.

### Supercells with oxygen Frenkel pair defects

**Fig 4:**
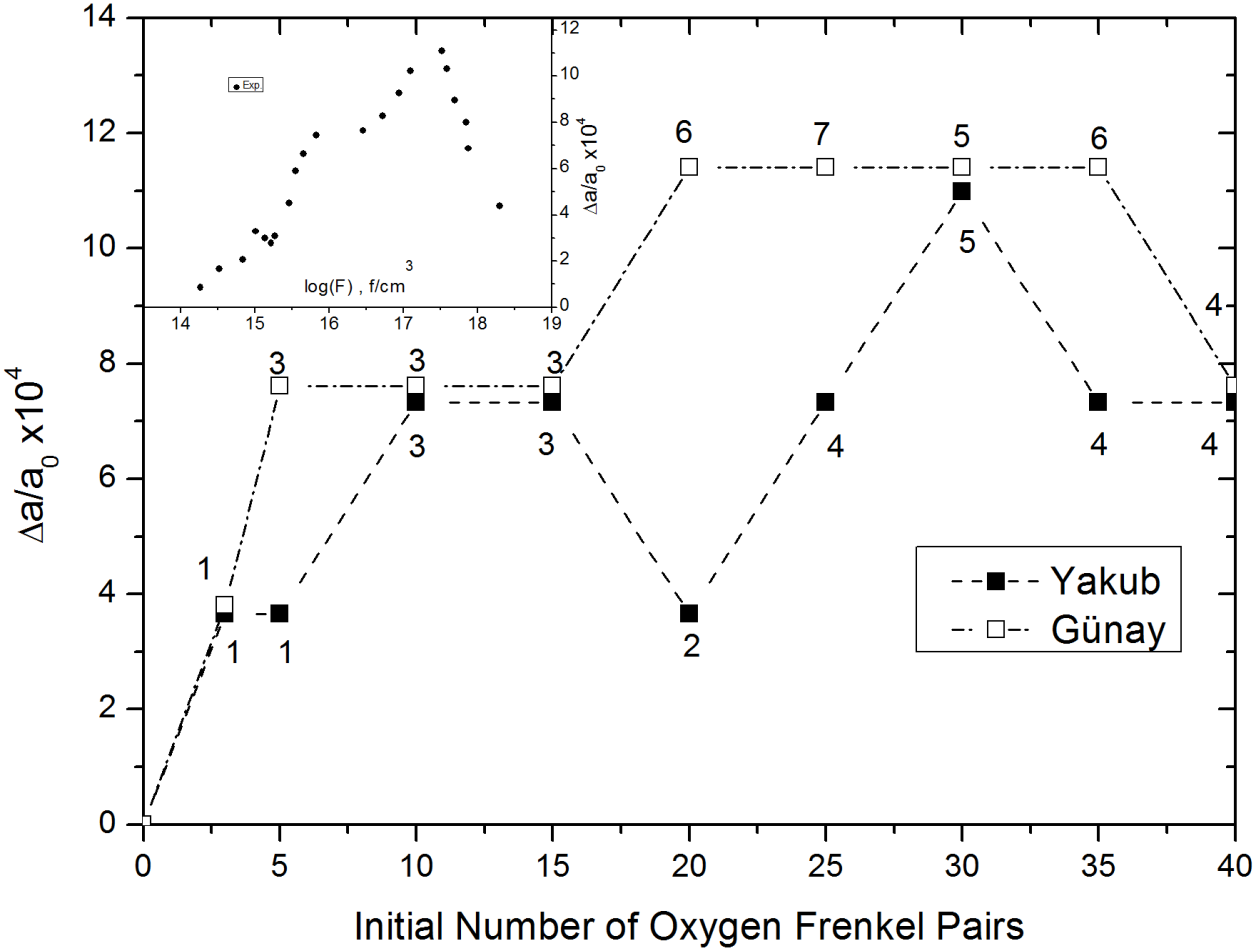
Lattice changes versus initial numbers of oxygen Frenkel pairs in MD supercells. The values given near the data points represent the numbers of obstruction type oxygen FPs in the MD supercell at the ends of simulations. The inset is taken from Ref. 17 and shows experimental data for lattice parameter changes that result from defect ingrowth as a function of increasing fission dose.

Fig 4 presents plots of the number of IFPs versus relative lattice expansion, obtained using Günay [1] and Yakub [5] potentials, and experimentally determined relative lattice expansion versus fission data from Matzke [17]. MD simulations of NPT ensembles were run using 10 different initially defected systems with numbers of oxygen IFPs varied from 0 to 40. Only some of the oxygen IFPs avoided recombination during the 10 ps equilibration step. Following equilibration, the numbers of FPs present, indicated by the numbers near the data points in Fig 4, remained constant. Three distinct stages have been experimentally observed to occur when fission dose is increased [17,18]: isolated FPs are produced at a constant rate (14<log(*F*)<16), then newly produced displaced atoms are trapped at existing defect sites, resulting in interstitial clusters (16<log(*F*)<17.5), and, finally, no more new interstitials are produced. This final stage is known as super saturation (17.5<log(*F*)<18.5).

These stages were successfully reproduced using both potentials. The maximum swelling points were estimated to be Δ*a*/*a*_*o*_ = 11.4×10^−4^–10.99×10^−4^ for Günay and Yakub potentials, respectively, which is very close to the experimental value, Δ*a*/*a*_*o*_ = 11.09×10^−4^. Based on simulation data, another stage appears to exist between 14<log(*F*)<15 which is not mentioned before [17,18]. Consequently, with this latest stage, the four stages are almost equidistant in the y direction and could be related to the diameter of space occupied by interstitial oxygen. In addition, relative lattice expansion is proportional to the number of surviving obstruction type oxygen FPs. Volume increments per obstruction type oxygen FP, Δ*v*_*F*_, were estimated from the following relationship [17]

$$\frac{\Delta a}{a_{o}}=\left(\frac{\Delta v_{F}}{3n_{cell}V_{o}}\right)N_{F}$$ (2)

where *n*_*cell*_ is the number of unit cells in the supercell, *V*_*o*_ is the unit cell volume, and *N*_*F*_ is the number of FPs. Δ*v*_*F*_ values of approximately 10 Å^3^ and 13.43 Å^3^ were obtained using the Günay and Yakub potentials, respectively. Nakea et al. [18] have estimated the volume increment of interstitials to be approximately 13.8 Å^3^ without indicating a specific sublattice (uranium or oxygen). However, in this work, it can be deduced that the change in the number of oxygen FPs (e.g. by fission irradiation) does not affect the uranium sublattice, which remains almost perfectly defect free. According to these observations, it can be concluded that experimental fission only creates oxygen FPs, which are responsible for lattice swelling.

### Supercells with uranium Frenkel pair defects

The aim of this section is to correlate the observed relative changes in the lattice parameter of both alpha-irradiated UO_2_ by experiment and the numbers of uranium FP defects predicted by MD simulations and to interpret those correlations. Uranium IFPs cause more defects than oxygen IFPs, therefore, this section has been prepared in two parts: one section presents the results of MD simulations run on 5×5×5 supercells, the other presents those run on 8×8×8 supercells. In order to obtain more consistent and reliable results, and determine the influence of defect images, the effects of varying periodic boundary conditions were observed by comparing the results obtained from 5×5×5 supercells containing uranium IFPs with those obtained from 8×8×8 supercells containing uranium IFPs.

### 5×5×5 supercells

**Fig 5:**
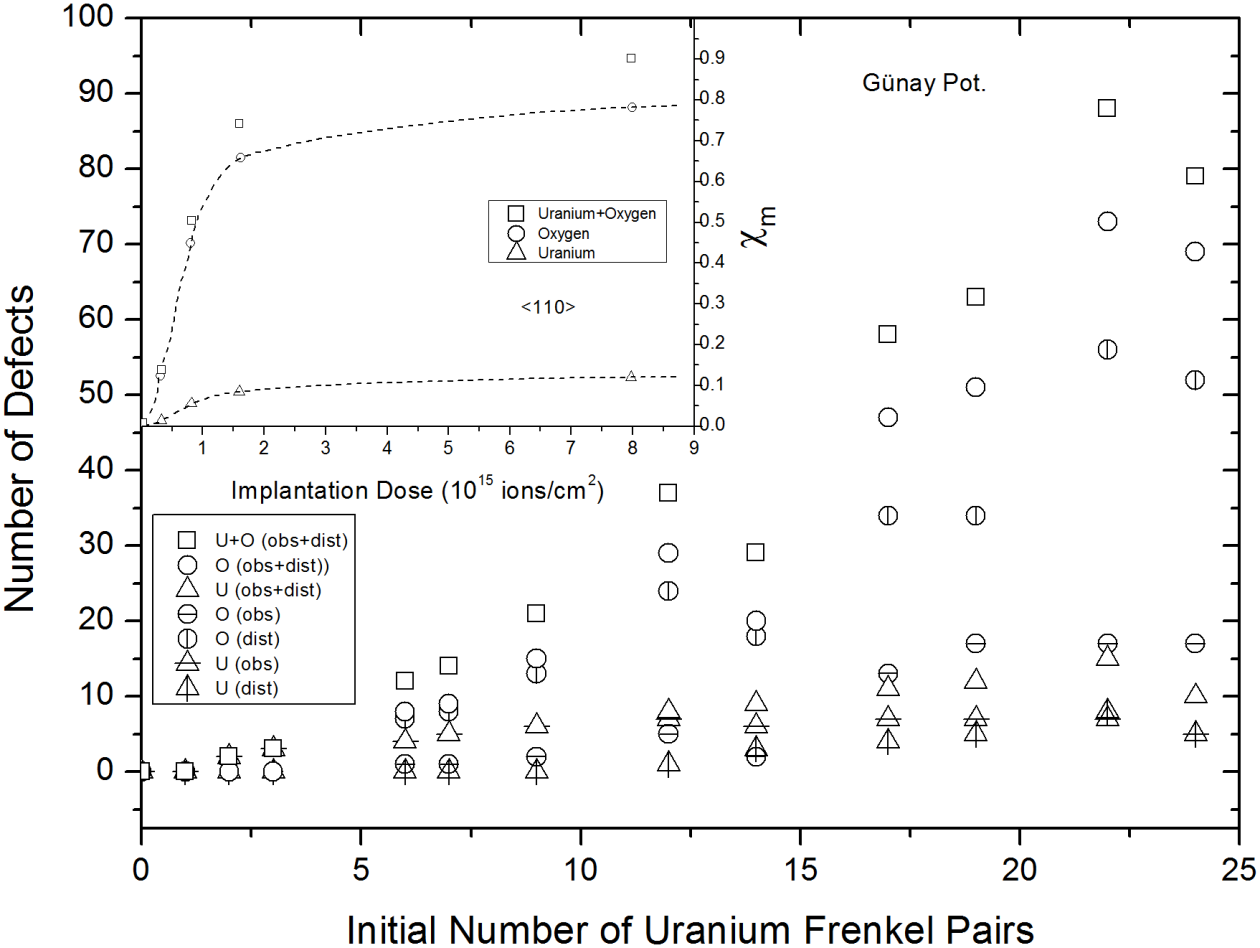
Günay potential results for variations in numbers of remaining and created FPs versus uranium IFPs. The inset was taken from Ref. 28 and shows the experimentally determined concentrations of uranium and oxygen FPs created, as functions of dose.

**Fig 6:**
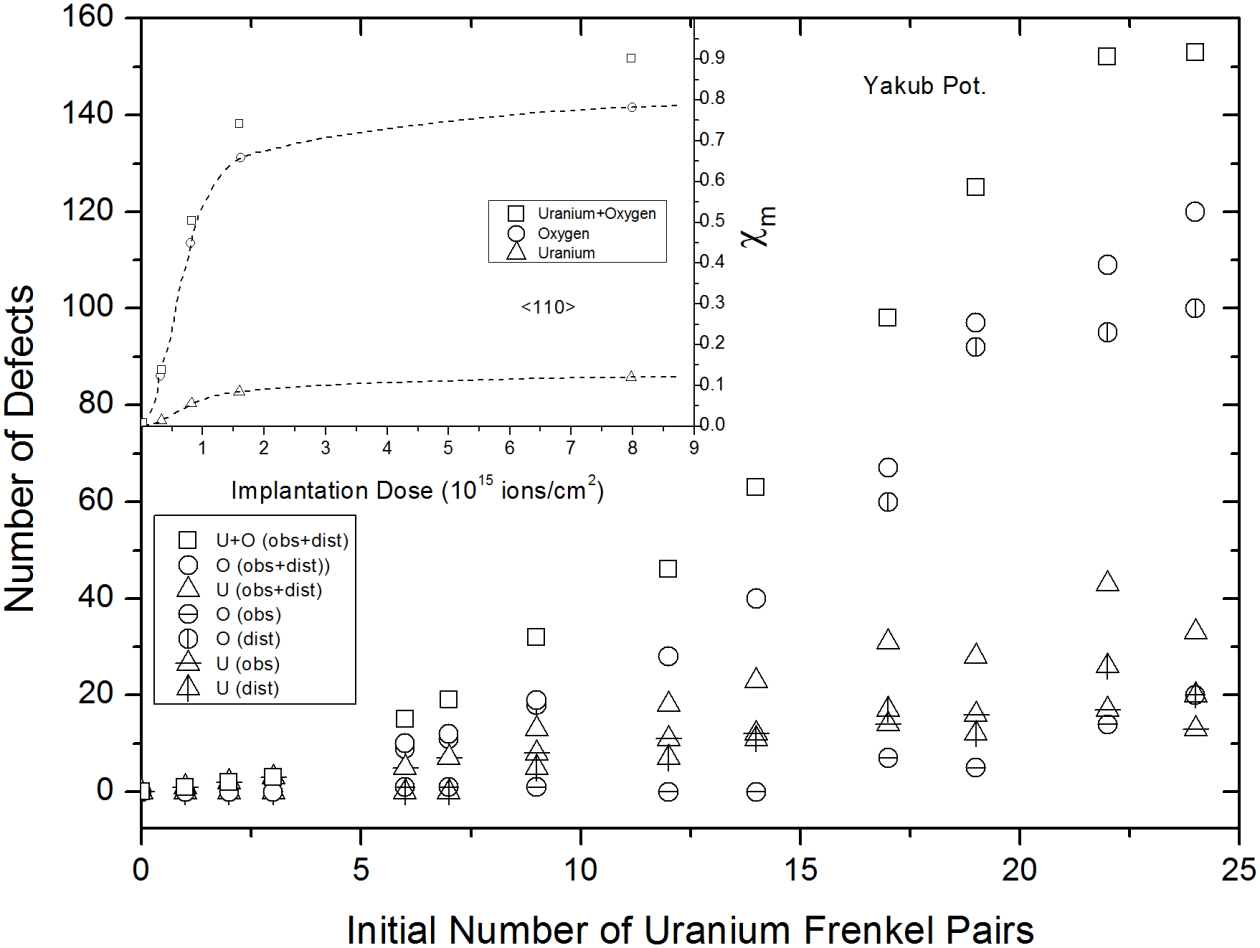
Yakub potential results for variations in numbers of remaining and created FPs versus uranium IFPs. The inset was taken from Ref. 28 and shows the experimentally determined concentrations of uranium and oxygen FPs created, as functions of dose.

The number of uranium IFPs was varied from 0 to 30, which was a large enough range to demonstrate the saturation stage using both Yakub and Günay potentials. In contrast to UO_2_ with oxygen IFPs, recombination of some of the uranium vacancies and interstitials during the equilibration step resulted in the creation of distortion and obstruction type uranium and oxygen FP defects. Figs 5 and 6 show how the number of surviving (remaining uranium IFPs) and created (uranium and oxygen) FPs varied with the number of uranium IFPs. These results were obtained from the <110> direction, as shown in Fig 2. The numbers of all types of defects increased as the number of uranium IFPs increased, eventually reaching saturation values. Distortion type oxygen defects were the most numerous and showed a sharp increase, quickly achieving a saturation value. A similar behavior was observed by Turos et al. [28] in their experimental study on radiation defects in UO_2_. For comparison, the insets in Figs 5 and 6 show how U and O defect concentrations vary with increasing implantation dose. This indicates that the large difference between the steering forces of U and O rows is responsible for the higher saturation value observed for oxygen ions than uranium ions. Steering force arises from differences in the masses and mobilities of U and O ions. It is known that O ions are naturally more mobile than U ions and that they have a liquid-like self diffusion coefficient at temperatures approximately 20% lower than the melting temperature of UO_2_ which is a property of a superionic conductor. Therefore, oxygen defects are more easily created during uranium IFP recombination, even at 300 K.

The experimental channeling yield ratio has been determined to be χ^*O*^/χ^*U*^≅6.7 [28]. Analogously, we estimate the ratio of the total number of defects (distortion and obstruction types) of oxygen ions to that of uranium ions to be about 3.3 using the Yakub potential and 5.4 using the Günay potential. Our results show that because of their larger effective ionic radii, *r*_*O*_/*r*_*U*_≅1.4, distortion type oxygen ion defects also partially block channels, even when not located at channel centers. This gives rise to the experimentally observed enhanced scattering yield [28].

Additionally, distinct features in Figs 5 and 6 indicate that further increases in uranium IFPs cause the numbers of obstructing and distorting uranium and oxygen ions to decrease. The tendency for defects to recover was not observed in experimental studies. This raises the question of whether, experimentally, further increasing the radiation dose would result in the partial recovery of defects and lattice contraction or not.

**Fig 7:**
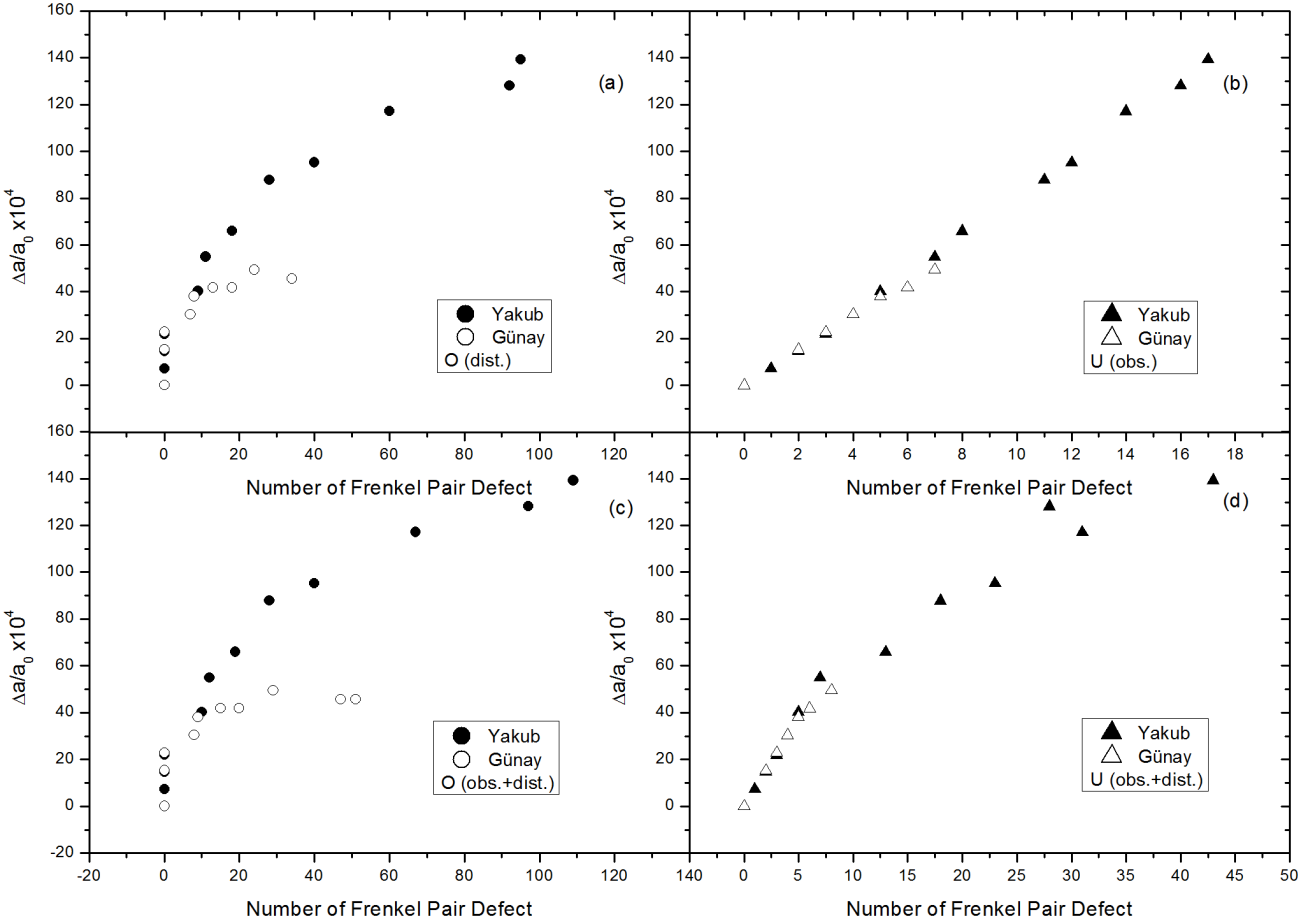
Relative lattice expansions of different types of defects: circles indicate oxygen, triangles uranium. (a) Oxygen distortions (b) uranium obstructions (c) oxygen obstructions + distortions and (d) uranium obstructions + distortions.

**Fig 8:**
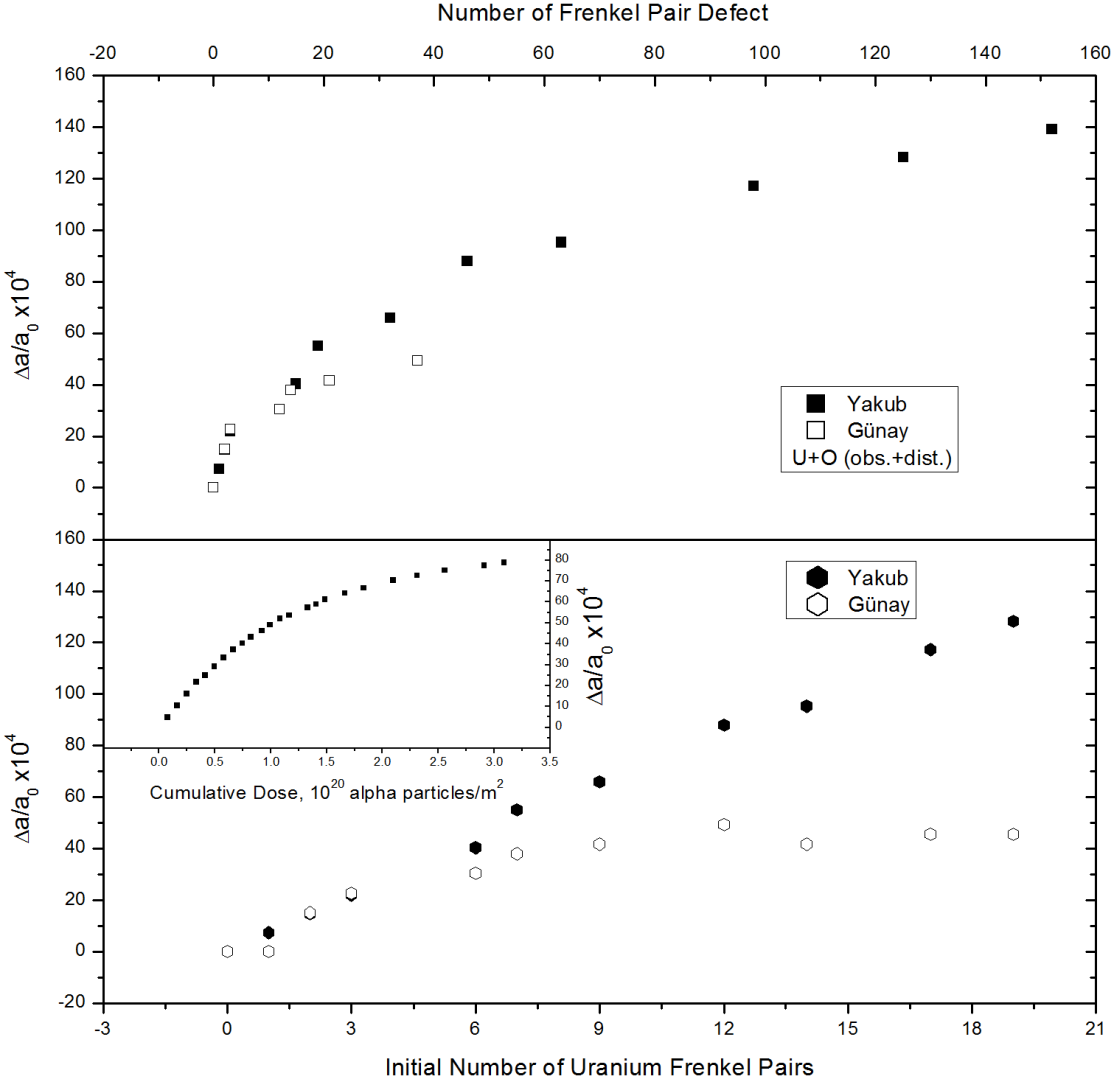
Relative lattice expansions. (a) Total number of defects after equilibration and (b) initial number of uranium FPs. The inset was taken from Ref. 21 and presents experimental lattice expansion data as a function of cumulative dose.

Figs 7 and 8 present plots of lattice expansion, Δ*a*/*a*_0_, versus number of defects. There is no clear functional relationship between Δ*a*/*a*_0_ and obstruction type oxygen defects or distortion type uranium defects. Obstruction type oxygen and distortion type uranium defects contribute almost nothing to the total number of defects (Figs 5 and 6) when present as IFPs in quantities of less than ~15 and ~10, respectively. Therefore, these defects have not been included in Fig 7. At lower than saturation levels, lattice expansion has an exponential dependence on the number of distortion type oxygen (see Fig 7(a)), distortion + obstruction types of oxygen (see Fig 7(c)), distortion + obstruction types of oxygen + uranium (see Fig 8(a)), and initial uranium FP (see Fig 8(b)) defects. The maximum lattice expansion observed was about 1.4% using the Yakub potential and 0.5% using the Günay potential. These values correspond to volume changes of 4.2 and 1.5%, respectively. For comparison, the experimentally determined relationship between lattice expansion and alpha dose, as determined by Weber [21], is shown as inset in Fig 8(b). Fitting the data obtained from the damage ingrowth model of Weber [21] yielded the expression Δ*a*/*a*_0_ = 8.4×10^−3^[1-exp(-0.85*D*_*α*_×10^−16^)], which predicts the lattice expansion to be 0.84% under saturation conditions [21]. Inspiring with Weber’s equation, we have also fitted the data to the same equation and in all cases the saturation value of the lattice expansion Δ*a*/*a*_0_ is estimated between 0.44%-0.48% for Günay and 1.3%-1.8% for Yakub potentials. The reason lower lattice expansion values were obtained using the Günay potential could be that it uses a stronger attractive interaction than the Yakub model does. Weber [21] has estimated that one defect pair for every 3–4 unit cells occurs in the saturation region and that such a defect concentration is indicative of isolated defects and negligible clustering. Both potentials resulted in very similar numbers of obstruction type defects to those determined by Weber. It is of interest that, as shown in Fig 7(b), lattice expansion varies linearly with the number of surviving obstruction type uranium defects. Evidently, such uranium defects, which are coordinated to six uranium ions, are responsible for lattice expansion. According to Eq 2, the gradient of the plot in Fig 7(b) indicates the volume increments per obstruction type uranium Frenkel pair, Δ*v*_*F*_, to be 39.56 and 49.47 Å^3^, using the Günay and Yakub potentials, respectively.

### 8×8×8 supercell

A similar procedure to that used with the 5×5×5 supercell was used with the 8×8×8 supercell. Up to 80 uranium IFP defects were included in the uranium supercell comprised of 2048 uranium ions and 4096 oxygen ions. An equilibrium run for every defected supercell was performed for 30 ps and data was collected for 70 ps. When the ions were visually analyzed, there was a clear distinction between the oxygen defects and the uranium defects. Uranium defects, which are generally obstruction type defects, have stable positions at octahedral interstitial sites and are coordinated with 6 other uranium ions in normal lattice positions. When the crystals were examined from the <110> direction it was clear that obstruction type uranium defects obstructed the channels, remained in their interstitial positions, and vibrated at their sites. In contrast, oxygen defects, which are generally distortion type defects, are mobile and have unstable interstitial positions. Such defects can move from one crystal position to another by crossing a channel or partially closing it (oxygen ions can be displaced into channels) and lack special positions. These conclusions can be deduced either from images or from the radial distribution functions of O-O and U-U.

**Fig 9:**
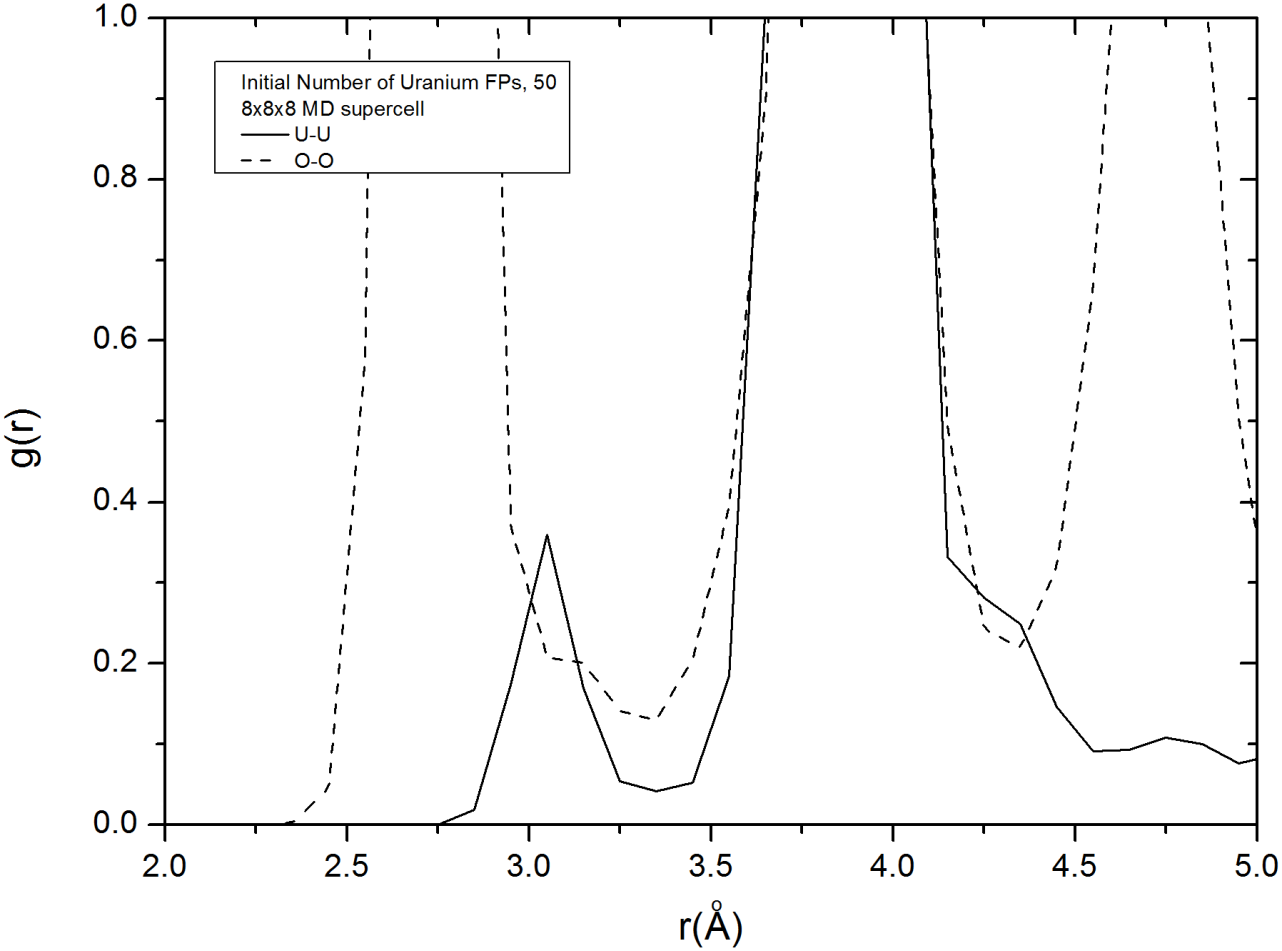
Radial distribution functions. *g*_*uu*_(*r*) and *g*_*oo*_(*r*) of UO_2_ with 50 uranium IFPs in a 8×8×8 MD supercell.

The plot of the radial distribution function obtained from the MD simulation, uranium interstitial defects are located between 2.75 and 3.4 Å from their surrounding uranium ions (Fig 9). Sharp peaks indicate immobile ions. Oxygen interstitials can be found between 3.2 and 3.4 Å away from other oxygen ions and show no significant peaks. Very few of oxygen interstitial ions are immobile and they could be observed from the <110> direction and were found to retain their positions within channels and were, thus, called obstruction type oxygen defects. Peaks associated with these oxygen defects are not present in Fig 9. We can assert that the peaks associated with the obstruction type oxygen defects could have merged with those associated with the distortion type oxygen defects. Obstruction type oxygen defects occur less frequently than distortion type oxygen defects and can only be observed at uranium IFP concentrations of 40 and greater.

**Fig 10:**
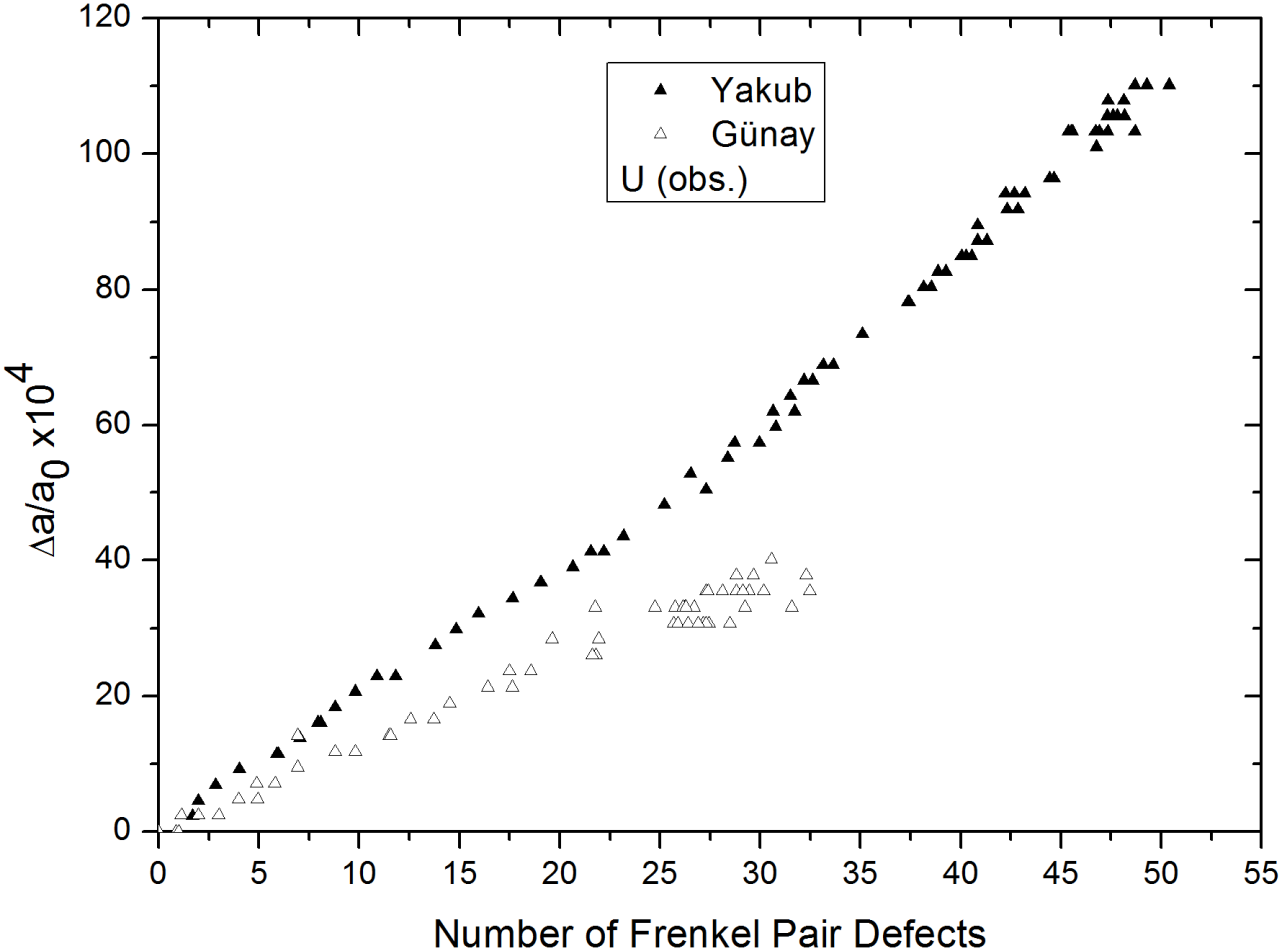
Relative lattice expansion versus number of obstruction type uranium defects, from Yakub and Günay potentials.

**Fig 11:**
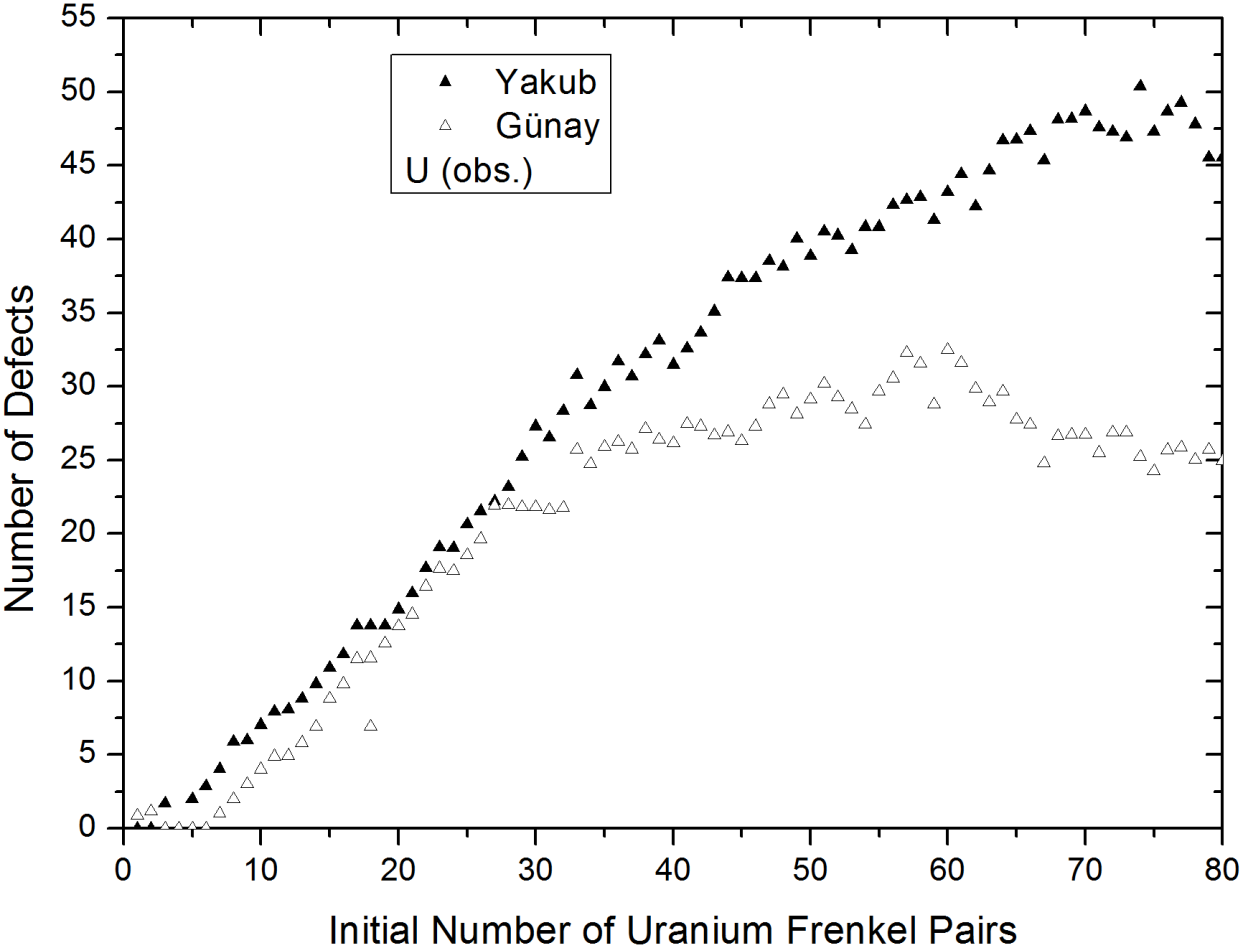
Number of uranium FPs versus IFPs, calculated using the Yakub and Günay potentials.

In Fig 10, lattice swelling clearly demonstrates a linear dependence on the number of obstruction type uranium defects for both potentials. This accounts for the lattice expansion observed during alpha particle irradiation. This was also observed in the 5×5×5 supercell, Fig 7(b), discussed in the preceding section and confirms the trend. Volume increments for obstruction type uranium FPs, as determined from Fig 10, are approximately 55.65 and 28.85 Å^3^ for the Yakub and Günay potentials, respectively. The value obtained from the Yakub potential is approximate because the slope of the plot in Fig 10 changes when the number of FPs exceeds 30. One possible reason for this is that obstruction type oxygen defects appear when more than 35–40 uranium IFPs, corresponding to 30 obstruction uranium FPs, are present, as seen in Fig 11.

In Fig 6, shown in the previous section, it was also observed that obstruction type oxygen FPs appear in the presence of more than 15 uranium IFPs. As mentioned in the previous section, obstruction type oxygen defects account for only a very small fraction of oxygen defects. From the slope of the Yakub potential graph shown in Fig 10, the volume increment was calculated to be 46.62 Å^3^ when 0–30 uranium FPs were present and 64.97 Å^3^ when 30–50 uranium FPs were present. The difference between these values is 18.35 Å^3^, which closely corresponds to the previously discussed, in this paper, volume increment value calculated for oxygen FPs using the Yakub potential, 13.43 Å^3^, and the published experimental value, 13.8 Å^3^ [18]. These observations indicate that a small number of oxygen defects penetrate into the octahedral cages of obstruction type uranium defects. It could be that some octahedral cages host one (An example is indicated with a blue arrow on right hand side of S1 Video. Uranium and oxygen ions are colored red and black, respectively.) or, less frequently, two oxygen atoms (indicated by a blue arrow on left hand side of S1 Video) and that such oxygen atoms vibrate at interstitial sites with obstruction type uraniums, acting as obstruction type defects. Not all obstruction type oxygen defects are involved in such formations. However, such a conformation, which can be observed visually, would explain the increased slope seen in Fig 10 when more than 30 obstruction type uranium FPs are present.

**Fig 12:**
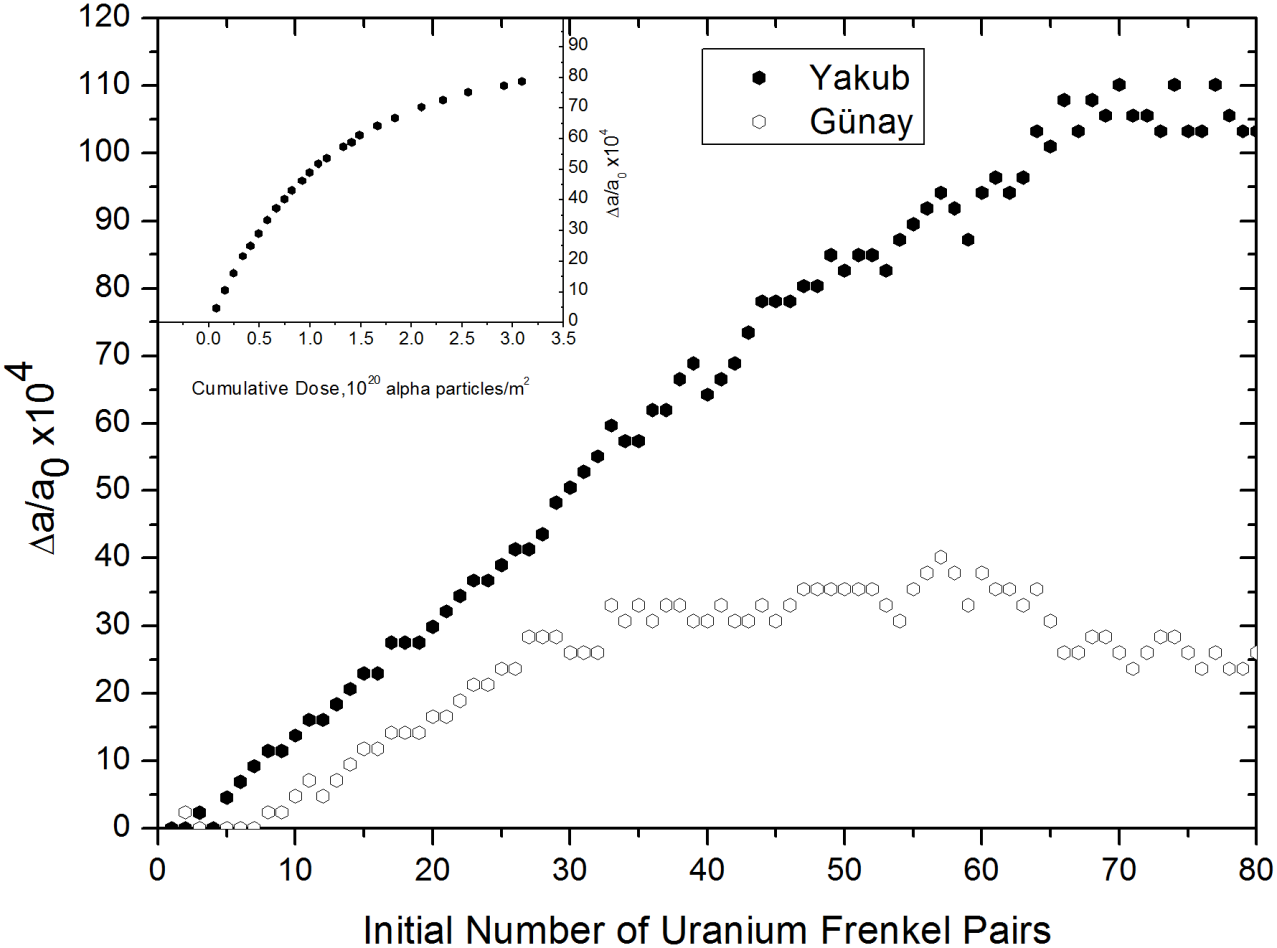
Relative lattice expansion versus number of uranium IFPs, calculated using the Yakub and Günay potentials. The inset was taken from Ref. 21 and shows experimental lattice expansion as a function of cumulative dose.

In Fig 12, obstruction type oxygen defects appear when ~35 to 80 uranium IFPs are present and lattice swelling decays exponentially, approaching a maximum, to saturation value. As the graph reaches a point of saturation value, data show a high variability. Here, octahedral cages of uranium or oxygen-uranium ions collapse at some point due to the enlargement of the lattice, which results in a decrease in lattice expansion. Moreover, reforming of these cages results in an increase in the lattice expansion, which turns out to be high-scattered data towards the saturation value in Fig 12. When obstruction type uranium ions are included in the supercell and obstruction type oxygen ions agglomerate around obstruction type uranium ions, lattice swelling reaches a saturation point and no space remains for obstruction defects. These saturation points were found to occur at 0.3–0.4% for the Günay potential and 1–1.1% for the Yakub potential. When the supercell size was increased from 5×5×5 to 8×8×8, the saturation value obtained from the Yakub potential agreed better with the experimental value, 0.84%.

## Conclusion

Molecular dynamics simulations were used to investigate how defect ingrowth affects lattice swelling in UO_2_, using two different partially ionized rigid ion potentials. Supercells with different defect concentrations were simulated under constant pressure and temperature conditions. It was found that some IFPs, in both samples with oxygen defects and samples with uranium defects, recombined during the equilibration step and induced additional obstruction and distortion type FPs. The number of induced defects in a sample remained nearly constant after equilibration.

The observed similarity between the lattice expansion that occurs as a result of oxygen defect ingrowth and that occurs as a result of fission dose indicates that fission damage can only create oxygen FPs and that they are responsible for lattice swelling. Experimentally observed lattice expansion stages and saturation values were successfully reproduced.

Simulation results for uranium IFP samples were similar to experimental data obtained in a study that examined defect production and annealing with high energy ^4^He ions [28]. Because of the large vibrations and sizes of oxygen ions, distortion type defects can also give rise to enhanced scattering yields even when oxygen ions are not occupying the exact centers of channels. Lattice expansion varies linearly with obstruction type uranium FP defects and exponentially with all other defect types. Six-coordinated (U-U) uranium interstitials cause lattice expansion. Although there are somewhat differences between the simulated and experimental lattice expansion saturation values, our estimated obstruction type defect concentration is comparable to the experimental value. This incident can be attributed to the MD simulation potentials which are over or under estimating the lattice constant. Both fission and alpha particle irradiation result in lattice expansion saturation because at some point the volumes of obstruction type ions are too big to be stable.

The next step in this work will be to study the effects of temperature on lattice recovery evolution using supercells with oxygen and uranium FPs. This will enable us to understand the effect of the sublattice on the recovery procedure.

## Supporting Information

S1 Video Equilibrium view of a UO_2_ supercell initialized with uranium IFPs, seen from the <110> direction. The blue arrow on the right hand side indicates a octahedral cage that is hosting one oxygen ion and one uranium ion. The blue arrow on the left hand side indicates two oxygen ions and one uranium ion. Uranium and oxygen ions are presented in red and black, respectively.
(MP4)
